# Clinician Misperceptions about the Importance of Adolescent HPV Vaccination

**DOI:** 10.4236/wjv.2016.61002

**Published:** 2016

**Authors:** Martin C. Mahoney, Frances G. Saad-Harfouche, Christy A. Widman, Annamaria Masucci Twarozek, Deborah O. Erwin, Elisa M. Rodriguez

**Affiliations:** 1 Department of Health Behavior, Roswell Park Cancer Institute, Buffalo, NY, USA; 2 Department of Cancer Prevention and Control, Office of Cancer Health Disparities Research, Roswell Park Cancer Institute, Buffalo, NY, USA

**Keywords:** Human Papillomavirus (HPV), Human Papillomavirus Vaccine, Adolescents, Cancer Prevention, Clinical Medicine

## Abstract

**Introduction:**

Adolescent HPV vaccination rates remain suboptimal. The purpose of the study was to investigate attitudes about HPV vaccine relative to other adolescent vaccines among clinical staff from primary care offices and school based clinics.

**Methods:**

We interviewed clinicians in primary care offices and school-based clinics regarding their attitudes about HPV vaccine relative to Tdap and MCV4.

**Results:**

Respondents (n = 36) included clinical staff in family medicine (47%), pediatrics (25%), obstetrics/gynecology (19%) and school-based health clinics (8%). Only 3% strongly agreed and 17% agreed that completion of HPV vaccine was more important than completion of pertussis vaccine (Tdap), while 6% strongly agreed and 33% agreed that completion of HPV vaccine was more important than completion of meningitis vaccine (MCV4).

**Discussion:**

Providing clinicians with additional information about the cancer prevention benefits of the HPV vaccine and the greater risk for HPV infection/disease relative to other vaccine preventable adolescent diseases may help to increase HPV vaccination rates among adolescents.

## 1. Introduction

According to national estimates, at least 79 million people in the United States (US) are presently infected with human papillomavirus (HPV), making HPV the most common sexually transmitted infection (STI) [1]. HPV includes more than 100 unique serotypes resulting in a variety of clinical outcomes ranging from abnormal pap smears to genital warts to cancers. Infection with one of ~ 15 “oncogenic” serotypes can lead to the development of cancers and precancers of the anogenital region and the oropharynx [2]. While most persons can clear acute infection without intervention, a subset of persons develop chronic persistent infections with cancer causing HPV types which can lead to malignancies. The International Agency for Research on Cancer (IARC) classifies oncogenic HPV serotypes as carcinogens [3].

Over 33,000 HPV-associated cancers are diagnosed annually in the US, with about 60% occurring among females; the proportion attributable to HPV is substantial including 90% of cervical and anal cancers, 70% - 75% of vaginal and oropharyngeal (base of tongue/tonsil) and 60% - 70% of vulvar and penile cancers [4] [5]. In addition to these invasive cancers, there are considerable numbers of HPV-associated high grade dysplasias involving the anogenital region, as well as abnormal pap smears resulting from HPV infection.

Three safe and effective vaccines are available to prevent HPV infection with the most prevalent oncogenic serotypes (e.g., 16 & 18). Gardasil^®^ (HPV4, quadrivalent vaccine against HPV types 6, 11, 16 & 18) was initially approved for use among females ages 9 - 26 years of age in 2006 to prevent cervical, vulvar and vaginal cancers/precancers and genital warts; the label was expanded in 2009 to include genital wart and anal cancer prevention in males ages 9 - 26. Cervarix^®^ (HPV2, bivalent vaccine against HPV types 16 & 18) was approved by the FDA in 2009 for protection against cancers and precancers in females [6] [7]. More recently, Gardasil 9 (9vHPV), which included coverage against 5 additional oncogenic HPV types (31, 33, 45, 52, and 58), was approved for use in females and males ages 9 through 26. In the US, Gardasil presently accounts for >95% of HPV vaccine sales.

Current recommendations from the Advisory Committee on Immunization Practices (ACIP) endorse HPV vaccination of both males and females at age 11 - 12, with catch up vaccination for females ages 13 - 26 and males ages 13 - 21 years; males aged 22 - 26 can also receive the vaccine [8] [9]. All HPV vaccines have demonstrated robust efficacy and no safety concerns [8]. Although reports of efficacy using a 2 dose schedule for both HPV2 and HPV4 vaccines have appeared, current recommendations in the US endorse a 3 dose series [8]. However, the impact of systematic education and programming by the Centers for Disease Control & Prevention (CDC) for instructions to clinicians about patient recommendation and recruitment to the HPV vaccine has been limited.

National estimates of HPV vaccination in the United States (US) (with either HPV2 or HPV4) reveal suboptimal coverage rates, which are substantially below the 80% target established by Healthy People 2020 and represent unrealized cancer prevention opportunities [10]. Among females ages 13 - 17 years, in 2014, 60.0% have received at least one dose and 39.7% have received all 3 doses. Comparable data for 2010 showed 48.7% with at least one dose and 32.0% with 3 doses consistent with modest increases over time [11]. Among males ages 13 - 17 years, in 2014, 41.7% have received at least one dose of HPV vaccine and 21.6% have received all 3 doses [11]. In contrast, uptake for Tdap and MCV4 vaccines (≥1 dose), also targeted to adolescents, are 87.6% and 79.5%, respectively [11]. Reasons for the disparity in completion rates for adolescent vaccines have included lack of provider recommendation, parental hesitancy, a general lack of urgency and missed opportunities to deliver HPV vaccine to adolescents at all office visits [10] [12].

In a national survey, fewer US physicians report HPV vaccination as important when compared to tetanus, diphtheria and acellular pertussis (Tdap) and meningococcal vaccines in an adolescent population. These clinicians also cited a need for longer patient counseling pertaining to HPV vaccine in contrast to other vaccines [13]. Another survey, also conducted among US physicians, found that less than two-thirds of pediatric and family medicine clinicians were making strong HPV vaccine recommendations for girls and even fewer were making the same recommendation for boys. Physicians who were less likely to discuss or endorse HPV vaccine felt that parents would not be receptive to HPV vaccine if offered [14].

This report investigates attitudes about HPV vaccine relative to other adolescent vaccines (e.g., Tdap and MCV4) among clinical staff from primary care offices and school based clinics.

## 2. Materials and Methods

Design: A mixed methods approach used structured interviews to understand/identify potential barriers and facilitators to adolescent HPV vaccination.

Population: Participants for the structured interviews were recruited through emails to primary care clinicians (Family Medicine, Pediatrics and Obstetrics & Gynecology [OB/GYN]) and members of a regional vaccine coalition across the western region of New York State (NYS). This analysis includes only respondents affiliated with primary care offices or non-college school-based health clinics.

Measures: Interviewer administered surveys lasting 25 - 30 minutes included closed-and open-ended items as well as self-reported demographics were conducted in 2015. A 5 point Likert scale (ranging from strongly agree to strongly disagree) was used to explore attitudes regarding 1) whether completion of HPV vaccine is more important than completion of MCV4, 2) whether completion of HPV vaccine is more important than completion of Tdap, and 3) whether completion of HPV should be required as is Tdap. Institutional Review Board approval was received for this project.

Statistical analyses: Descriptive analyses were conducted using SPSS 21.0 (©IBM, Armonk, NY).

## 3. Results

As shown in Table 1, respondents (n = 36) were predominantly female (n = 23, 64%), between 41 - 60 years of age (n = 27, 75%) and white (n = 29, 81%) and included clinical staff affiliated with family medicine (n = 17, 47%), pediatrics (n = 9, 25%), OB/GYN (n=7, 20%) and school based health clinics (n = 3, 8%); 61% (n = 22) were physicians, 17% (n = 6) advanced practitioners and 17% (n = 6) nurses.

**Figure 1:**
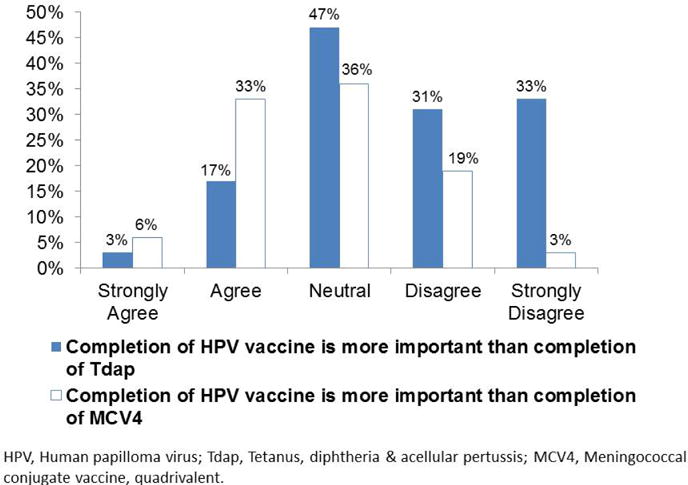
Attitudes regarding importance of HPV vaccine to Tdap and MCV4 vaccines among adolescents.

As presented in Figure 1, just 3% strongly agreed and 17% agreed that completion of HPV vaccine is more important than completion of Tdap, while 6% strongly agreed and 33% agreed that completion of HPV vaccine is more important than completion of MCV4. Tdap is a school mandated vaccine in NYS for students 11 years of age or older entering grades 6 through 12 while similar requirements will be implemented for meningitis vaccine in September 2016; 19% of respondents strongly agreed and 28% agreed that HPV vaccinations should also be mandatory.

## 4. Discussion

As it relates to results of this study, two recent reviews have examined barriers to HPV vaccination. A US based study noted that clinicians expressed concerns about parent attitudes regarding vaccination while parents reported knowledge gaps and lacked a valid understanding of disease risk versus benefits of vaccination; parents however, did endorse the importance of clinician recommendation in having their child vaccinated [12]. A second review which included international publications, although predominantly based upon US studies, suggested that an adolescent female’s access to HPV vaccine is influenced by national health policy (e.g., vaccination recommendation and availability), perceived social norms and values, recommendations from clinicians, and parental willingness to have their children vaccinated [15].

Although physician recommendation is considered to be an essential component of getting vaccinated [16], clinicians may lack a comprehensive knowledge of the benefits of HPV vaccination, have inadequate skills to communicate that information and/or may attempt to make a determination regarding “risk behaviors” by adolescents as well as “parental hesitancy” in deciding which patients to recommend for HPV vaccination, or may deliver a weak recommendation [17]-[19]. As a result, clinician perception influences what occurs during the clinical encounter. Moreover, reasons commonly reported by parents of adolescents not vaccinated against HPV include lack of a recommendation from their physician, concerns about safety/side effects, limited knowledge about HPV and the vaccine, and an impression that their adolescent is not sexually active [10] [17].

Our findings suggest that another contributor to the lack of urgency for HPV vaccination may be misperceptions among clinicians regarding both the risk of HPV infection and severity of HPV-related disease relative to both pertussis (prevented by Tdap vaccine) and bacterial meningitis (prevented by MCV4 vaccine). It could be argued that the array of cancers and pre-cancers associated with HPV infections [20] is more compelling than either self-limited pertussis infections (25.1/100,000 among adolescents) [21] or meningococcal disease (0.3 - 0.5/100,000) [22], which although clinically severe, is far less common. It is possible that clinicians providing care to adolescents may commonly encounter pertussis or even meningitis but be less familiar with the incidence and seriousness of HPV-associated disease occurring among adults.

Similar to the theme identified in our study, a recent report noted comparable findings based upon a different research design [13]. The study by Gilkey *et al*. (2015), used an online survey of pediatricians and family physicians who were recruited through a survey research company; the response rate was 43% (1022/2368) and 76% of respondents (n = 776) were eligible and completed the survey. Nearly 2/3 of respondents described a standardized approach to presenting vaccines to their 11 - 12 year old patients with 73% presenting Tdap vaccine first, compared to meningococcal vaccine (15% first) and HPV vaccine (12% first); conversely, 70% of the time HPV vaccine was discussed last. This suggests that clinicians approach HPV vaccine differently from other recommended adolescent vaccines and it is possible that parents may perceive this hierarchical approach as ascribing less importance to HPV vaccine. Relatedly if physicians are perceived as strongly endorsing completion of Tdap and/or meningococcal vaccines [23] [24], parents may identify a relatively more modest endorsement of HPV vaccine as indicative of weaker clinician support [13].

School vaccine mandates have been successful in decreasing disease incidence and increasing rates of vaccination [25]. Despite misperceptions of disease risk attributable to HPV, when compared to vaccination against pertussis and meningitis, 47% of respondents in this study agreed that HPV vaccine should also be a school requirement; Tdap is currently mandated for NYS students age 11 and attending 6^th^ grade while meningitis vaccine will be required starting in September 2016. While a school mandate would be expected to increase rates of HPV vaccination [26], the level of potential resistance by parents, communities, and health care professions, as well as the impact of “opt-out provisions”, make it difficult to predict the result of this approach in our state.

These findings have strong clinical relevance since they are based on the engagement of a group of participants providing clinical care to adolescents. Limitations of this study include its focus on a single geographic region and enrollment of a limited number of participants using a non-random recruitment process. This approach could have resulted in respondent bias favoring the delivery of HPV vaccination. However study results do not support such an enrollment bias, and if present, the true results would likely be even less enthusiastic regarding the importance of HPV vaccination relative to other adolescent vaccines.

## 5. Conclusion

In conclusion, only 20% and 39% of participants agree or strongly agree that completion of HPV vaccine is more important than completion of Tdap or MCV4 vaccine, respectively. This observation may explain in part reasons why many clinicians seem to prioritize completion of adolescent vaccines other than HPV. These results suggest a potential opportunity to review with clinicians the epidemiology and disease burden attributable to HPV, relative to pertussis and meningitis, as an additional strategy to correct misperceptions and to systematically recommend HPV vaccines to 11 - 12 year olds.

## Figures and Tables

**Table 1:** Selected demographic characteristics of study participants (n = 36).

Characteristics	Number	%
**Gender**		
Female	23	63.9
Male	13	36.1
**Age**		
18 – 30	1	2.8
31 – 40	3	8.3
41 – 50	15	41.7
51 – 60	12	33.3
>60	5	13.9
**Race/Ethnicity**		
Non-Hispanic White	29	80.7
Hispanic, Any Race	1	2.7
Non-Hispanic, Other Races	5	13.9
Decline	1	2.7
**Type of Practice**		
Family Medicine	17	47.3
Pediatrics	9	25.0
Obstetrics & Gynecology	7	19.4
School Based Health Clinics	3	8.3
**Professional Title**		
Physician	22	61.1
Nurse Practitioner/Physician Assistant	6	16.7
Registered Nurse/Licensed Practical Nurse	6	16.7
Other	2	5.5
